# Qualitative Analysis of Caregiver and Patient Experiences With and Barriers to Medical Nutrition Therapy Utilization in Pediatric Type 1 Diabetes

**DOI:** 10.1016/j.eprac.2025.11.005

**Published:** 2025-11-21

**Authors:** Svetlana Azova, Hannah Michelson, David Williams, Belinda Lennerz, Katharine Garvey

**Affiliations:** 1 Division of Endocrinology, Boston Children’s Hospital, Boston, Massachusetts; 2 Department of Pediatrics, Harvard Medical School, Boston, Massachusetts; 3 Biostatistics and Research Design Center, Boston Children’s Hospital, Boston, Massachusetts

**Keywords:** medical nutrition therapy, pediatric, qualitative study, semi-structured interviews, type 1 diabetes

## Abstract

**Objectives:**

Despite recommendations by major diabetes organizations, medical nutrition therapy (MNT) in pediatric type 1 diabetes (T1D) remains underutilized. Suboptimal prandial practices and limited engagement in nutritional counseling with the diabetes care team may impact health outcomes in these youth. This study aimed to understand patient and caregiver experiences with and barriers to MNT utilization.

**Methods:**

Utilizing the socioecological framework and the phenomenological research design augmented by grounded theory methods, we conducted semi-structured interviews with caregivers of patients with T1D < 18 years and youth with T1D 12–17 years with T1D duration ≥1 year. Emergent themes were used to generate a theoretical model of the relationship between the family unit encompassing the child with T1D and suboptimal MNT utilization.

**Results:**

We recruited 9 caregivers and 9 patients. Qualitative analysis yielded important themes highlighting challenges impacting MNT utilization across 5 domains: *individual*, *shared child/caregiver-specific*, *interpersonal*, *institutional*, and *community/societal*. The central theme was recognition of the importance of MNT in pediatric T1D management juxtaposed with barriers to its implementation. Factors mediating this paradoxical relationship included the impact of diabetes technologies on prandial practices, balance between caregiver involvement and emerging patient independence, emphasis on normalcy, attempts to match child’s way of eating with that of others, school-based considerations, and cultural/sociodemographic influences.

**Conclusions:**

This study generated informative insights on the perceptions and experiences of patients and caregivers regarding barriers to MNT implementation in pediatric T1D. Efforts to enhance MNT appreciation and utilization in this population by targeting the identified barriers through patient-/family-centered strategies are crucial.

## Introduction

Major diabetes organizations, including the American Diabetes Association (ADA) and International Society for Pediatric and Adolescent Diabetes, acknowledge the importance of individualized medical nutrition therapy (MNT) in children with type 1 diabetes (T1D) for glycemic and weight management and in the prevention of long-term complications.^1,2^ However, many children and families encounter challenges with optimal MNT utilization,^3–10^ despite documented glycemic^6,10–13^ and metabolic^5,7,14^ benefits. This is often manifested by suboptimal dietary quality^3,4,6,8^ and low engagement with a registered dietitian (RD).^9,10^ Prior studies identified several potential barriers. These included general concerns about time constraints, expense, child food preferences, and picky eating, and themes unique to diabetes, such as caregiver desire to avoid limiting their child’s diet or making them feel different^15,16^ and difficulties with carbohydrate estimation.^17^ Culturally-based nutritional challenges have also been identified among Hispanic caregivers of children with T1D.^18^

Most studies cited above have focused on assessing children’s adherence with the recommended RD follow-up or dietary composition from a caloric and macronutrient standpoint or evaluated caregivers’ perceived challenges to compliance with those guidelines.^3,4,6,8–10,15,17,18^ Qualitative research offers a unique opportunity to analyze the voices and lived experiences of participants to more deeply understand attitudes, beliefs, and expectations about care and ultimately inform intervention design. Our qualitative study involving separate semi-structured interviews with patients and caregivers of children with T1D is innovative in utilizing the socioecological framework^19^ to understand perceptions of MNT and barriers that may be contributing to MNT underutilization. We aimed to use our findings to build a theoretical model of factors impacting MNT utilization in pediatric T1D. Ultimately, understanding the drivers of and barriers to MNT utilization will facilitate design of interventions aimed at improving health outcomes in this population.

## Methods

### Study Design, Setting, and Selection of Participants

We utilized a phenomenological research design augmented by grounded theory methods^20^ to investigate drivers of and barriers to optimal MNT utilization in pediatric T1D. This hybrid research design allowed us to utilize the socioecological framework^19^ to explore the lived participant experiences with MNT and to identify knowledge gaps in our understanding of factors contributing to MNT underutilization. We assessed MNT utilization via a combination of prandial practices and engagement in nutritional counseling with the diabetes care team. Optimal prandial practices were based on whether the child’s reported way of eating generally met the benchmarks for a healthful diet^1,2^ and how well youth adhered with the recommended mealtime insulin administration practices (ie, pre-bolus dosing and carbohydrate/insulin matching).^1,2^ Engagement in nutritional counseling was assessed via whether participants had incorporated nutrition-focused discussions into routine clinical visits with their diabetes care team (eg, if the child had an RD visit in the electronic medical record [EMR] within the last 12 months of enrollment).

We conducted separate 1:1 semi-structured interviews with patients and caregivers of children with T1D followed by the Outpatient Diabetes Program at Boston Children’s Hospital (BCH), a large, pediatric, tertiary care, urban, academic medical center that serves approximately 2200 children with diabetes. All participants had a single study visit. Inclusion criteria were English-speaking caregivers of patients with T1D < 18 years and youth with T1D 12–17 years with T1D duration of ≥ 1 year and ≥ 1 diabetes medical visit at BCH in the past year. In addition, the participant had to be able to participate in the interview (eg, verbal, without prohibitive cognitive or developmental delays).

Eligible participants were identified from an Outpatient Diabetes Population Management database used for quality improvement and from review of the EMR. Initially, participants for each group (ie, caregiver and youth with T1D) were randomly selected from the database in blocks of 30 and maximum variation purposive sampling strategies were employed to recruit participants with a diverse distribution of characteristics of interest. These patient-related characteristics included age, sex, diabetes duration, use of a continuous glucose monitor (CGM), mode of insulin delivery, most recent hemoglobin A1c (HbA1c) (within the last 12 months prior to the date of random selection), and presence/absence of an RD visit within 1 year prior to the date of random selection. Email and telephone outreaches were conducted by trained study personnel. To aid in recruitment, we also approached several endocrinologists and diabetes nurse educators for potential referrals. Lastly, we posted study flyers at the BCH diabetes clinics and on the private BCH Diabetes Program Facebook and Instagram pages. If the same patient appeared in both cohorts on random selection, a team decision was made to only keep them on one list based on purposive sampling needs. However, if referred by the medical team, or contact was self-initiated, considerations for enrollment of both the child and caregiver were made on an individual basis, if combined interest was expressed. If a caregiver-patient dyad was enrolled for separate interviews, the interviewers performed 1 interview each prior to any analysis to avoid bias. As recruitment was ongoing, theoretical sampling was employed in response to emergent themes during analysis until data saturation was reached. All participants received $75 compensation.

### Data Collection

To facilitate data collection, detailed interview guides (Supplemental Files 1 and 2) were developed covering 4 major topics: (1) general questions about the child’s T1D history, (2) general thoughts about the role of nutrition in a child’s T1D care, (3) patient-specific nutrition-related questions, and (4) experiences with nutrition-focused discussions and recommendations encountered during visits with the diabetes dietitian/nutritionist and medical providers (this data will be explored in a separate manuscript). The interview guides were developed by the research team, with consultation from faculty with expertise in pediatric T1D and qualitative research. While the guides contained prompts covering a comprehensive overview of topics to capture the needed data, interviewers were instructed to utilize a semi-structured style to provide deeper insights.

Two trained female interviewers (S.A., H.M.) conducted the semi-structured interviews between October 2023 and April 2024. Interviews were carried out virtually using the Zoom platform (Version 5) and lasted an average of 60 minutes. Interviews were audio- and video-recorded and transcribed using Hoffman Transcription. Interview transcripts were reviewed and edited for accuracy prior to analysis. In addition to the recordings, interviewers kept an observation log for each interview, writing down pertinent participant verbal comments and nonverbal cues and behaviors, and engaged in reflexivity reflections^20^ post-interview, documenting overall impressions and thoughts about emergent themes.

### Data Analysis

Descriptive statistics were calculated and presented as means and standard deviations (after confirming normality with Shapiro-Wilk test) or proportions, as appropriate.

For qualitative analysis, the research team initially utilized a combination of inductive and deductive coding approaches and ultimately condensed their findings into categories and themes. Prior to coding, a multidisciplinary research team of pediatric endocrinologists, experts in qualitative research, and a research assistant created a draft codebook informed by the socioecological framework.^19^ Emergent coding was also utilized based on data from the first 6 interviews. The actual analysis consisted of 3 phases, led by the same 2 study team members who conducted the interviews (S.A., H.M.). In the first phase, each coder read the finalized interview transcript and then completed independent, blind level 1 coding of the interviews. After coding of an interview was complete, coders met and reviewed the applied codes, resolving any differences. The codebook was iteratively amended based on notes and observations from the first 6 coded interviews (3 caregiver, 3 patient). Those interviews were later recoded using the updated codes. Additional coders (K.G., D.W.) were available to resolve any discrepancies and disagreements in the coding.

In the second phase, after identifying a gap in our understanding of the exact drivers of MNT underutilization in pediatric T1D, the study team invoked grounded theory methods and engaged in axial coding to expand upon the core concepts and to connect the level 1 codes into broader categories. In the final phase, team members met to identify major themes and to explore the interconnections between these in order to build a theoretical model of factors mediating and impacting the relationship between the family unit encompassing the child with T1D and MNT utilization. Throughout this iterative process, we modified our recruitment efforts via theoretical sampling strategies to capture additional insights and observations needed to refine aspects of our emergent model that required further clarification. Data collection and analyses were completed when theoretical saturation was achieved. Throughout all phases, the coders engaged in analytic memo writing to generate additional insights and inform axial coding, higher level analysis, and acknowledgment of reflexivity.^20^ All analyses were performed using Dedoose software (Version 9.0.107, SocioCultural Research Consultants, LLC).^21^

### Data Availability

The datasets generated during and analyzed in the current study and the coding dictionary used are available from the corresponding author upon reasonable request.

## Results

### Participant Characteristics

**Fig. 1.**
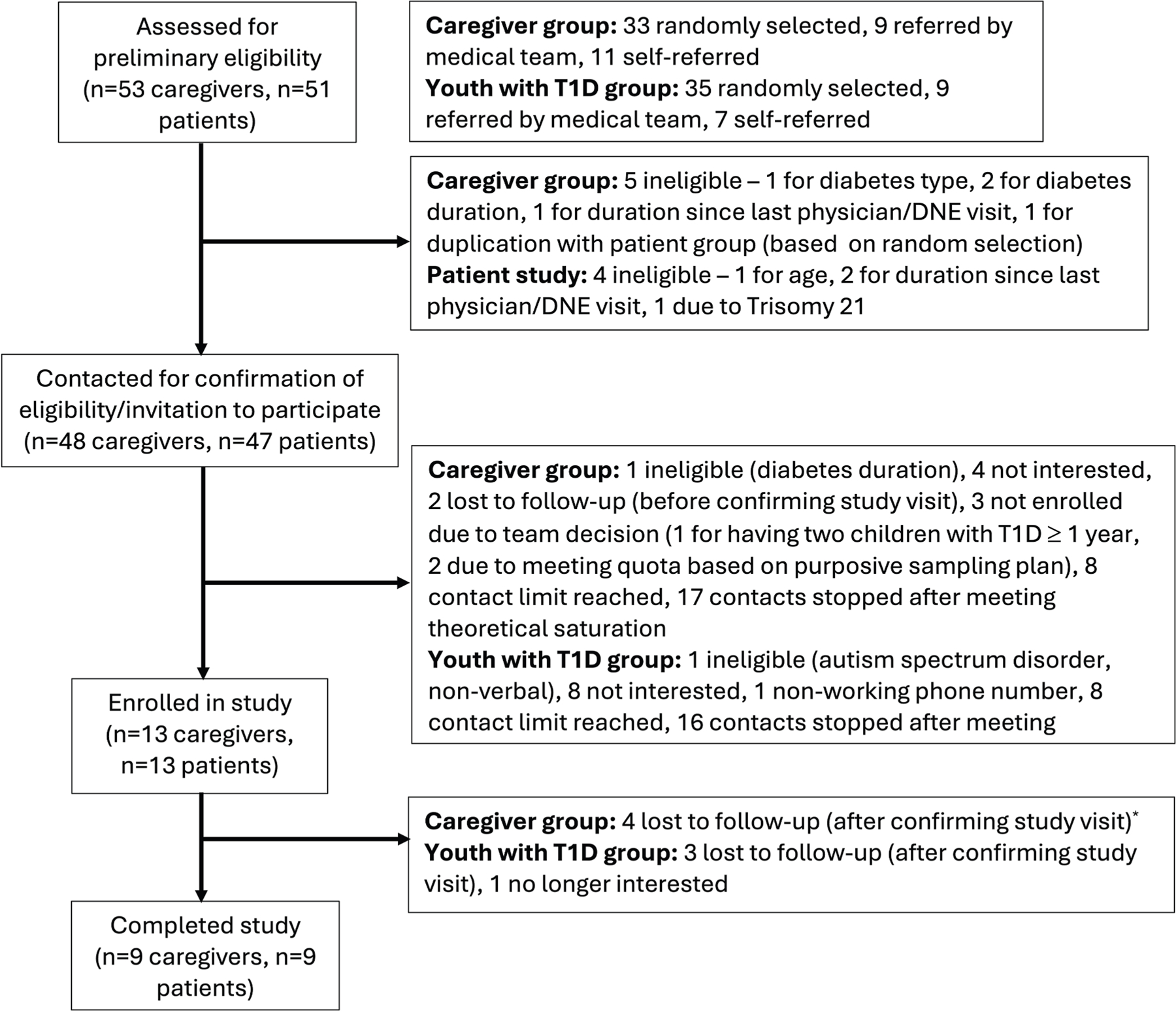
Flow chart of participant recruitment for the qualitative study. *DNE* = diabetes nurse educator; *T1D* = type 1 diabetes. *Under participants lost to follow-up after confirming study visit in the caregiver group, 2 of the children on behalf of whom the interviews were intended to be conducted were Black, non-Hispanic, and one was Hispanic.

A total of 18 participants were included in the study (9 caregivers, 9 patients) (Fig. 1). There was only one caregiver-patient dyad that participated in the respective interviews, both self-referred. For the caregiver group, we present relevant characteristics for both the caregivers and the children on behalf of whom the interviews were conducted (Table 1). Almost all caregivers were female (88%), all were white, non-Hispanic, and completed more than 12 years of education, and the majority were in the higher-than-average income bracket. The average age of the children on behalf of whom the interviews were completed was 12.0 ± 5.2 years, 67% were female, and the majority had favorable HbA1c values and were using a CGM and automated insulin delivery (AID) system (similar to the statistics seen in our Diabetes Program). One child had celiac disease. Child characteristics in the youth with T1D group are summarized in Table 2. The average age of the youth was 15.5 ± 1.6 years, 56% were female, and the majority were using a CGM and AID system. Notably, only 33% of children in both cohorts had a single one-on-one RD visit in the prior year.

### Qualitative Analysis

**Fig. 2.**
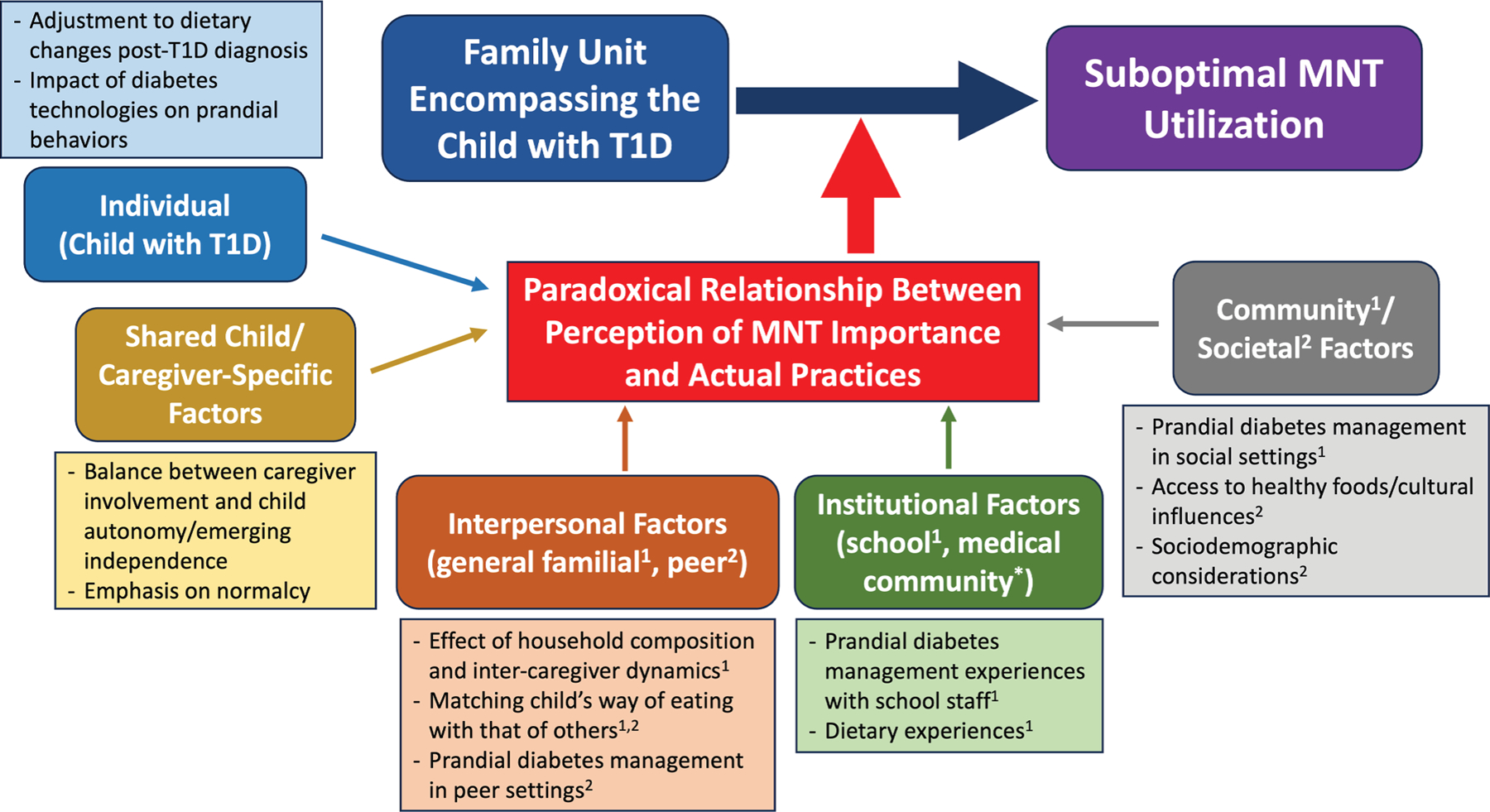
Theoretical model of factors impacting the relationship between the family unit encompassing the child with T1D and MNT utilization. *MNT* = medical nutrition therapy; *T1D* = type 1 diabetes. *Experiences within the medical community will be explored in detail in a separate manuscript.

Our iterative analysis identified several key themes addressing drivers of and barriers to optimal MNT utilization in pediatric T1D that covered the 5 main interconnected domains, informed by the socioecological framework^19^: *individual*, *shared child/caregiver-specific*, *interpersonal*, *institutional*, and *community/societal*. The themes within each domain are presented below, supported by salient participant quotes. These themes were then used to generate a theoretical model depicting the intricate process that connects the family unit encompassing the child with T1D and suboptimal MNT utilization (Fig. 2). The central theme that emerged was a paradoxical relationship between appreciation of MNT importance and actual practices, with several emergent mediating themes, described below.

### Domain 1: Individual (Child) Factors

Both caregivers and patients identified several individual (child) factors influencing the relationship between the family unit encompassing the child with T1D and MNT utilization. The quotes associated with each theme are presented in Table 3.

#### Theme 1: Difficulties with Adjustment to Dietary Changes Post-Diabetes Diagnosis.

Participants described challenges that children with T1D experienced when adjusting to diet-related changes post-diagnosis, which not uncommonly led to restricted eating behaviors in the child, sometimes also due to fear of or discomfort with insulin injections. Conversely, several participants described maladaptive strategies aimed at regaining a sense of food freedom and normalcy, including sneaking of food and using hypoglycemia as a catalyst. Factors that seemed to play a protective role in helping to facilitate adjustment included child age at T1D diagnosis, pre-diabetes eating habits, and the presence of routine/structure.

#### Theme 2: The Impact of Diabetes Technologies on Way of Eating and Prandial Behaviors.

Caregivers and patients described associations between diabetes technologies and maladaptive prandial behaviors in the child, including intake of foods of lower dietary quality due to a sense of increased food freedom and de-intensified prandial diabetes management practices. Concurrently, several participants noted benefits of diabetes technologies in facilitating prandial management.

### Domain 2: Shared Child/Caregiver-Specific Factors

The following themes reflect factors relevant to both the child with T1D and the caregiver. The quotes associated with each theme are presented in Table 4.

#### Theme 1: Balance Between Caregiver Involvement and Child Autonomy/Emerging Independence When it Comes to the Child’s Nutritional Management.

Some participants described challenges with regard to emerging independence and balancing responsibility for the management of the child’s prandial behaviors, especially when it came to adolescents. The ability to effectively balance caregiver involvement and child autonomy through a collaborative approach was often associated with more optimal nutritional practices in the patient.

#### Theme 2: Relationship Between Acknowledgment of Optimal Prandial Management and its Execution.

Both patients and caregivers, while acknowledging the importance of optimal prandial management, identified several general challenges related to its execution.

Many participants acknowledged challenges with (1) prandial insulin timing and (2) carbohydrate/insulin matching (the latter in some cases attributed to fear of hypoglycemia). Many saw deviations from the expected practices as an adaptation for glycemic stability based on the current situation or prior experiences/observations. Regardless, participants often acknowledged the importance of these prandial practices.
(3) Both caregivers and patients described management nuances and difficulties specific to various components of the meal/macronutrient composition (ie, differential effects of carbohydrates, glycemic index, protein, fat, and portion sizes on blood glucose) and foods that are challenging from a glycemic perspective. Some participants presented strategies for managing the differential effects of meal/macronutrient composition and challenging foods. Others gave examples of foods that had a more favorable effect on the child’s glycemic trends, often in association with a lower insulin requirement. Many participants also described observed/perceived effects of dietary factors on health beyond glycemia.
(4) There were several factors driving approaches to the child’s way of eating, including emphasis on maintaining normalcy/not restricting. In addition, while none of the children with T1D in this study had a history of a diagnosed eating disorder, concern for the development of disordered dietary habits was not uncommon. These sentiments may have contributed to some participants describing the child’s overall diet quality as being suboptimal or variable. Many participants, however, also acknowledged the importance of mindful eating habits, translating into healthful practices in the child. When asked to assess their general understanding and comfort when it came to managing the child’s way of eating, participants often described these favorably. However, some noted fluctuations in their understanding and comfort or acknowledged that these alone were not always sufficient in optimizing MNT utilization.

#### Theme 3: Juxtaposition Between Perception of Overall Importance of Nutrition in T1D Care and Desire for Normalcy.

In addition to actual practices, overall participant perceptions about the role and importance of nutrition in T1D care also impacted MNT utilization in the child. The emphasis was once again on maintenance of normalcy and prioritization of diabetes management, although several participants expressed the general perception of the importance of making mindful choices when it comes to food options/practices and acknowledged the role of individualization. When asked about the overall importance of nutrition, many participants recognized the pivotal role that it plays both in diabetes management (including when compared to other treatment modalities) and overall health, amidst challenges balancing recognition and actual practice.

### Domain 3: Interpersonal Factors

There were several general familial and peer-related factors impacting the relationship between the family unit encompassing the child with T1D and MNT utilization. The quotes associated with each theme are presented in Table 5.

#### Subdomain 1: General Familial Factors

##### Theme 1: Effect of Household Composition and Intercaregiver Dynamics on Approaches to the Child’s Way of Eating.

Nontraditional household composition and divergent approaches to way of eating presented challenges to the child’s nutritional management. Conversely, an intact household and convergent views on nutritional management were often protective factors when it came to optimal MNT utilization.

##### Theme 2: Attempts at Matching Child’s Way of Eating with That of the Rest of the Family.

Participants described challenges matching the family’s way of eating to that of the child with T1D. However, some caregivers shared the family’s successful attempts at supporting and integrating the child’s way of eating into their routine, despite potential challenges. Baseline familial dietary habits preceding the child’s T1D diagnosis at times facilitated this adjustment.

#### Subdomain 2: Peer-Related Factors.

In addition to dietary challenges related to the family unit, participants also described peer-related factors that have impacted the relationship between the child with T1D and MNT utilization. These included difficulties surrounding matching the child’s way of eating with that of the peers (Theme 1) and challenges with prandial diabetes management in peer settings (Theme 2).

### Domain 4: Institutional Factors

Participants described school-related challenges that may have impacted the relationship between the child and MNT utilization. The quotes associated with each theme are presented in Table 6.

#### Theme 1: Variable Prandial Diabetes Management Experiences with School Staff.

Several participants acknowledged staff-related challenges when it came to the child’s prandial management at school. However, some participants endorsed more positive, collaborative interactions between the patient/family and school staff.

#### Theme 2: Suboptimal School-Related Dietary Experiences.

Both caregivers and patients also described specific diet-related challenges at school, often related to food quality. Some participants endorsed both (1) self-/family-directed and (2) school-related accommodations that facilitated the child’s nutritional management.

### Domain 5: Community/Societal Factors

Broader community and societal factors also played an important role in mediating the relationship between the family unit encompassing the child with T1D and MNT utilization. The quotes associated with each theme are presented in Table 7.

#### Subdomain 1: Factors Related to Broader Community/Social Settings.

Participants described variable experiences with prandial diabetes management in social settings (Theme 1). Several noted challenges with proper insulin timing and carbohydrate/insulin matching (eg, due to the unpredictability of the meal composition at restaurants). Others felt self-conscious about insulin administration in public. Despite challenges, some participants endorsed adaptive prandial strategies in the social setting, including the availability of nutritional information in restaurants. A few emphasized the value of community support and education in helping to manage the child’s way of eating.

#### Subdomain 2: Societal Factors.

Larger societal factors also played a pivotal role in mediating the relationship between the family unit encompassing the child with T1D and MNT utilization. These included nutritional challenges stemming from easier access to foods of lower dietary quality, often in the setting of cultural influences (Theme 1), and potential impact of sociodemographic inequities on MNT utilization (Theme 2).

## Discussion

Our qualitative analysis of semi-structured interviews elucidated valuable insights regarding experiences with MNT among both patients and caregivers of children with T1D and identified important barriers to MNT utilization using the socioecological framework. There was a central theme juxtaposing participant recognition of the importance of MNT in the management of the child’s T1D with challenges to its actual implementation, mediated by factors across 5 domains (*individual*, *shared child/caregiver-specific*, *interpersonal*, *institutional*, and *community/societal*). These challenges to MNT implementation included difficulties with adherence to a healthful way of eating, appropriate timing of prandial insulin administration, and proper carbohydrate/insulin matching and suboptimal engagement in nutritional counseling with the child’s diabetes care team (eg, absence of an RD visit within the prior year). Prior studies highlighted the emphasis on normalcy and avoidance of dietary restriction,^15,16^ as well as inherent challenges with carbohydrate estimation,^17^ as potential barriers. While our study offered further credence to these concepts, it expanded our understanding of the pivotal role that the child’s surrounding environment plays in ensuring optimal MNT utilization, underscoring the need for diabetes medical providers to consider and address factors that both encompass and extend beyond the child-caregiver dyad (eg, those related to the family unit as a whole, peers, school setting, and broader community) through patient-/family-centered strategies.

Among individual (child-related) factors, difficulties with adjustment to dietary changes post-T1D diagnosis often contributed to challenges with optimal MNT utilization, even amidst acknowledgment of its importance. Adjustment to a new, lifelong diagnosis is challenging, especially if it is associated with the need for multiple adaptations and changes to one’s previous lifestyle habits and routines. This applies to youth with T1D,^16^ which ultimately impacts their overall perceptions about and experiences with MNT. As demonstrated in our study, maladjustment to prandial changes can lead to food avoidance, which may increase future risk of disordered eating behaviors. These are relatively common in youth with T1D^22^ and should be screened for regularly during visits with the diabetes care team.^2^ Conversely, some children exhibited loosened dietary practices, including sneaking of food and using hypoglycemia as a catalyst for food freedom (particularly for high-carbohydrate items). While overtreatment of low blood glucoses in youth with T1D has been described, at times attributed to the fear of hypoglycemia,^23^ to our knowledge, the concept of using it as a way to regain a sense of normalcy has not been commonly reported.

Protective factors that facilitated adjustment to nutritional changes included younger age, baseline healthy eating habits, and presence of routine/structure. Patients that were diagnosed at a younger age tended to have an easier adaptation to nutritional changes, as these simply became part of their identity and felt just like normal events/persistent parts of life. In addition, children who had followed more healthy eating habits pre-diagnosis also had an easier adjustment to diet-related modifications following T1D diagnosis. Finally, presence of routines and schedules has previously been recognized in helping to facilitate diabetes self-management in youth with T1D.^24^ It is thus not surprising that adherence to routines set by families often helped optimize MNT utilization in the child, and deviations from set practices created both anticipated and unanticipated challenges. These factors are important to consider when approaching patients and families of children with T1D in order to appropriately tailor nutritional counseling to meet individual needs.

When it comes to diabetes technologies, and especially AID systems, the themes of increased food freedom and relaxed mealtime bolus dosing practices were prevalent in our study, often resulting in increased consumption of higher glycemic index foods, larger portion sizes, inconsistent pre-bolus dosing, and imprecise carbohydrate/insulin matching. While these devices have revolutionized the care of children with T1D, resulting in improved overall glycemic outcomes, reduced risk of hypoglycemia, and decreased disease burden,^25–27^ prior studies have suggested that their use may in fact be associated with the aforementioned maladaptive prandial practices^28–30^ and delivery of higher insulin doses,^27^ at least partially in response to post-prandial glycemic excursions. This can lead to both increased intake of lower quality foods and resultant excessive weight gain.^31^ While the idea of increased food freedom may certainly have benefits, especially in ameliorating disease burden and if restricted eating was previously a concern, access to foods of lower quality and improper prandial insulin management may have pronounced negative long-term consequences, especially on cardiometabolic health. Thus, concrete efforts should be made to properly educate patients using diabetes technologies, particularly AID systems, to continue to be mindful of their dietary choices and pay careful attention to proper prandial diabetes management.

This study also uncovered several shared themes among child and caregiver-specific factors impacting MNT utilization in youth with T1D. One recurring theme was the balance between caregiver involvement and emerging independence in the child with regard to nutritional management. It was evident from many interviews that with increasing independence, many children struggled to make healthy eating choices or engage in optimal prandial diabetes management practices. Older age was also recently identified as being associated with a lower likelihood of annual RD follow-up among children with T1D.^10^ The ADA acknowledges that self-management in pediatric diabetes should involve both youth and their caregivers, emphasizing the importance of family involvement through childhood and adolescence,^2^ which has been associated with positive outcomes when it comes to treatment adherence.^32^ With regard to nutritional management in T1D, several studies have shown that caregiver choices and perceptions impact youth diet quality.^33,34^ However, maintaining a balance between interdependence and independence can be challenging, especially in adolescence. This can certainly impact children’s prandial behaviors, as demonstrated in our study. A recurring protective factor in attaining an effective balance between caregiver involvement and patient independence in this study was the use of a collaborative approach and involving the child in the decision-making process, even starting at a young age, which may result in healthier nutritional choices. These concepts are important to consider when providing dietary counseling to families across different child age ranges and transitional stages.

Within the interpersonal domain, general familial factors also impacted the relationship between the child with T1D and MNT utilization. Among them was the effect of household composition and inter-caregiver dynamics on approaches to the child’s way of eating. Presence of nontraditional households without 2 intact caregivers may be associated with distinct struggles when it comes to all aspects of managing a chronic disease, including T1D, especially in the setting of concurrent sociodemographic challenges.^35^ In these cases, competing demands may force attention to be focused on the most critical aspects of care (ie, insulin management). Additionally, if more than one household is present, variability in approaches is possible, as described by several participants in this study, leading to inconsistencies in prandial management. The same may also apply to intact households where caregivers hold different perceptions regarding dietary practices. Conversely, convergent caregiver approaches and positive modeling behaviors are important factors in facilitating healthy eating habits in the child.^33,34^ Thus, these factors should be assessed for and incorporated into any nutritional counseling that occurs for youth with T1D and their families.

Related to both general familial and peer-related factors, participants also discussed challenges in matching the child’s way of eating with that of their family members and peers. When it comes to families, those with baseline mindful dietary practices typically had an easier time with integration. With regard to peers, the concept of trying to fit in and the impact of peer perceptions on diabetes self-management have been described in literature.^36,37^ In some studies, the presence of peer support seemed to have a positive impact on the emotional well-being and treatment adherence in adolescents with T1D,^32,37^ something that also came out in this study. These concepts underscore the importance of both assessing for possible general familial and peer-related challenges impacting diabetes self-management in youth and disseminating information on T1D to the community and schools, as well as empowering children and adolescents with this disease to provide appropriate education to their family members and peers.

Within the domain of institutional factors influencing the relationship between the family unit encompassing the child with T1D and MNT utilization, for this manuscript, we decided to highlight school-related considerations and challenges. Participants described both staff- and diet-related challenges impacting the child’s ability to optimally manage their way of eating and prandial behaviors at school. Some participants did identify both self-/family-directed and school-related accommodations, including bringing food from home and availability of carbohydrate counts for meals, respectively. However, the latter was not universally present, thus creating the potential for inequities in the children’s ability to properly manage prandial aspects of their diabetes at school. Both the ADA and other prior studies acknowledge the importance of proper diabetes management in schools, including providing appropriate training and support to the personnel responsible for the care of the child and ensuring access to the necessary nutritional information, which may ultimately improve patient outcomes and experiences.^2,38,39^ Public health advocacy efforts to improve the overall food quality at schools for all children are also important. The diabetes care team should regularly assess for these potential school-related barriers to optimal MNT utilization and advocate on behalf of their patients’ needs.

While not discussed within the scope of this manuscript, interactions within the medical community undoubtedly shape the way families and children with T1D approach MNT. It is notable that only 33% of participants in both cohorts had a single one-on-one RD visit within the year prior to enrollment. While low, this is higher than the average annual RD follow-up rate among youth with T1D recently identified at our institution (20.8%).^10^ Regardless, limited engagement with this important component of the diabetes care team can certainly impact participant perceptions and experiences regarding MNT and will be explored further in a separate analysis.

Lastly, but importantly, broader community and societal factors also played a role in how families and children with T1D approached MNT utilization. There was an aspect of unpredictability regarding available food options, carbohydrate content, and/or proper insulin management in social settings (eg, restaurants). In addition, some children felt perceived judgment and shame from others with regard to prandial insulin management in public. The latter has previously been described in the context of general challenges with diabetes self-management in social settings among youth with T1D.^36,37^ When it comes to societal influences, many called out the stereotypical American diet as generally not being the most healthful^40^ and described challenges with figuring out insulin doses to cover the carbohydrate content of foods without nutrition labels. Preference for prepackaged versus whole foods was previously described in T1D, due to the ease afforded by carbohydrate estimation for the former.^17^ High availability of and easy access to energy-dense foods/snacks, often leading to a lower diet quality in the child, may complicate glycemic management and increase future risk of cardiovascular disease in patients with T1D. Many participants in our study expressed desire for additional counseling on how to properly manage foods without nutrition labels.

Several caregivers also acknowledged that healthier foods tend to be more expensive. In one study, the presence of severe food insecurity in patients with type 2 diabetes was associated with higher body mass index in the setting of lower overall caloric consumption but intake of energy-dense foods with higher glycemic load.^41^ There is limited data on this topic in T1D,^42–46^ with documented associations between the presence of food insecurity and a higher HbA1c in youth with T1D,^42,46^ potentially mediated at least in part by the former’s impact on MNT.^44^ Cultural, racial, and ethnic challenges often have an impact on overall diet quality among individuals and may certainly affect both diabetes management^35^ and MNT utilization in youth with T1D.^18^ As an example, a recent study exploring the lived experiences of Hispanic caregivers of children with T1D followed by our institution identified culturally-based nutritional challenges, including difficulties with acculturation and integration, especially within the context of the T1D diagnosis, as well as uncertainty of how to incorporate medical advice into their dietary habits.^18^ Careful consideration of sociodemographic and cultural factors is imperative in providing individualized, equitable nutritional care to youth with T1D and their families.

This study has several limitations. The small sample size and lack of diversity may have limited the transferability of our findings. However, the comprehensiveness of the data collection within the context of the socioecological framework,^19^ the inclusion of both caregivers and patients, and the use of maximum variation purposive and theoretical sampling strategies allowed us to gather wide-ranging and representative insights regarding experiences with and barriers to MNT utilization among youth with T1D, thereby achieving data saturation. Nonetheless, we were unable to explore all potential barriers to MNT utilization in depth. For example, only one child in the study (caregiver group) had celiac disease, which presented unique insights, however, did not allow us to perform a comprehensive analysis. In addition, many children, particularly in the caregiver group, had favorable HbA1c values compared to the most recent average HbA1c in our overall diabetes population (8.0% ± 1.6%),^31^ missing opportunities to effectively understand how barriers to MNT utilization may impact prandial management of youth struggling to meet glycemic targets. Thus, future studies can focus on additional challenges related to MNT utilization in patients with certain comorbid conditions and those with higher HbA1c levels.

In addition, for the purposes of this study, we did not consider patient race and ethnicity or caregiver socioeconomic status and educational level under purposive sampling criteria, the former due to the availability of some recently published data (particularly for Hispanic youth followed by our program^18^) and the latter because of the logistical and ethical challenges in determining these at the time of recruitment and enrollment. All of the patients in our study identified as White, non-Hispanic (in our program, ~75% are White, non-Hispanic, ~5% Black, non-Hispanic, and ~10% Hispanic) and most caregivers in the caregiver cohort were in the middle-income bracket or higher and all completed 12+ years of education, suggesting a particularly motivated group and introducing selection bias. When it comes to race and ethnicity, while we did intend to recruit a more diverse sample, it is notable that for the caregiver group, 3 out of the 4 participants lost to follow-up after confirming the study visit had children that were either Black, non-Hispanic or Hispanic. This suggests potential unique challenges these families may face when it comes to participation in virtual interviews, despite the study team’s attempts to accommodate their specific situations (eg, agreeing to do the interviews in the evening or over the weekend). While our qualitative findings are likely not representative of the patient population as a whole, this study still generated important pilot data on MNT utilization perceptions and barriers, paving the road for the design of larger studies to capture the insights of families of children with T1D who come from historically marginalized backgrounds and/or have lower socioeconomic status and educational level in the caregiver(s).

Lastly, the potential for interviewer bias and power differential should be considered. Both interviewers made efforts to build rapport and ensure participant comfort (eg, emphasizing participant confidentiality and avoiding wearing a white coat), while maintaining professional distance, to ensure data validity. Researcher biases were continually examined through the use of reflexivity,^20^ engaging in reflective analytic memo writing following each interview and during every stage of the analysis.

## Conclusions

In conclusion, this qualitative study of children and caregivers of youth with T1D provided an important opportunity to explore their experiences with MNT and to understand potential barriers to its optimal utilization. A socioecological framework was employed to characterize the conveyed challenges into *individual*, *shared child/caregiver-specific*, *interpersonal*, *institutional*, and *community/societal* domains. The central theme was the overall recognition of the importance of nutritional considerations in the management of the child with T1D juxtaposed against barriers to their implementation in real-life scenarios. In addition to providing useful preliminary data to inform broader investigative studies, we hope that our findings will help motivate the design of future interventions aimed at promoting MNT appreciation and utilization and improving both short- and long-term health outcomes in children with T1D.

## Supplementary Material

MMC2

MMC1

## Figures and Tables

**Table 1:** Caregiver Group Participant Characteristics

	Caregiver completing study Mean ± SD or *n* (%)	Child on behalf of whom study is being completed Mean ± SD or *n* (%)
Age (y)	43.6 ± 7.5	12.0 ± 5.2
Female sex	8 (88%)	6 (67%)
Race and ethnicity		
White, non-Hispanic	9 (100%)	9 (100%)
Educational level		N/A
More than 12 y^a^	9 (100%)	
Income bracket		N/A
$45,000-$59,999	1 (11%)	
$60,000-$75,999	1 (11%)	
$75,000-$99,999	1 (11%)	
$100,000+	6 (67%)	
Diabetes duration (y)	N/A	4.8 ± 3.4
CGM use	N/A	9 (100%)
Mode of insulin delivery	N/A	
MDI		1 (11%)
Insulin pump without automation		1 (11%)
AID system		7 (78%)
Hemoglobin A1c (%) [mmol/mol]	N/A	6.7 ± 0.8 [49.4 ± 8.5]
RD visit in the past year	N/A	3 (33%)
Comorbid health conditions	N/A	
Anxiety/depression		1 (11%)
ADHD		1 (11%)
Celiac disease		1 (11%)
Hypothyroidism		2 (22%)

*Abbreviations:* ADHD = attention-deficit/hyperactivity disorder; AID = automated insulin delivery; CGM = continuous glucose monitor; MDI = multiple daily injections; RD = registered dietitian; SD = standard deviation.

a Defined as more than high school graduate or equivalent, including associates degree, some college, or college degree.

**Table 2:** Youth Group with Type 1 Diabetes Participant Characteristics

	Child characteristics Mean ± SD or *n* (%)
Age (y)	15.5 ± 1.6
Female sex	5 (56%)
Race and ethnicity	
White, non-Hispanic	9 (100%)
Diabetes duration (y)	5.8 ± 3.4
CGM use	9 (100%)
Mode of insulin delivery	
MDI	3 (33%)
AID system	6 (67%)
Hemoglobin A1c (%) [mmol/mol]	7.8 ± 0.8 [61.7 ± 8.0]
RD visit in the past year	3 (33%)
Comorbid health conditions	
Anxiety/depression	2 (22%)
ADHD	1 (11%)

*Abbreviations:* ADHD = attention-deficit/hyperactivity disorder; AID = automated insulin delivery; CGM = continuous glucose monitor; MDI = multiple daily injections; RD = registered dietitian; SD = standard deviation.

**Table 3:** Individual (Child) Factors Impacting the Relationship Between the Family Unit Encompassing the Child With Type 1 Diabetes and Medical Nutrition Therapy Utilization

Theme 1: Difficulties with adjustment to dietary changes post-diabetes diagnosis
Development of restricted eating patterns
7P ^a^ : *I quickly learned that food was associated with shots, so I would often refuse to eat, and I would even like eat without people knowing, or like I would just like try my hardest to like not get the shot, and it would lead to either me not eating or just like eating like in secret.*
9P: *When I first got diagnosed with diabetes,… I stopped eating as much sugar because so many people were telling me that was going to solve my problems if I stopped eating sugar, that it would make my blood sugar go down all the time, so I kind of stopped eating sweets, but it made me so miserable that I just started eating more sweets again…*
Maladaptive strategies to regain sense of food freedom/normalcy
5P: *I’ve always been somebody who generally has a pretty large appetite and does enjoy, you know, carb-heavy stuff, and so when my blood sugar’s low, it feels like the only time I can actually kind of eat like a normal person again, so it makes me just eat a ton of carbs…*
7P: *…after I got diagnosed…, and I realized how diabetes works,… I would sometimes jump my way home from school…, so by the time I got home, my sugar was low, and I could get candy, so I wasn’t really thinking about like healthy things. I was just trying to think of ways I could get candy back…*
Protective factors
(1) Age at T1D diagnosis:
1P: *I mean I don’t really remember [how I’ve changed my diet since receiving the diagnosis], but I know it’s just like being able to eat whatever I want. That’s never been a thing for me because I was so young, I don’t even remember it…*
(2) Pre-diagnosis eating habits:
2C ^a^ : *For my family, we didn’t have juice in our house. We didn’t eat candy. My kids had never gone to McDonald’s or Burger King. Like that was just not part of our family culture of who we were, so for us, there wasn’t a huge shift minus the gluten-free [for concurrent celiac disease in the child].*
(3) Presence of routine/structure, deviations from which may lead to nutritional challenges:
5C: …*we have a schedule, we have a routine, okay, she ate cake out of her routine, so she’s a little bit thrown off, so like her body’s learned the schedule, so I think anything that’s out of the schedule throws her off anyway. You know, if lunch is half an hour late, she’s like, “I need all of the food in my face all at once,” you know, so I think the schedule is important.*
6P: …*when I get on more like a schedule of what I eat and it kind of is just like consistent like every day, I feel like it’s kind of cumulative, and my blood sugars are much better like when I get more in the routine of that. Like during the holidays, just like Thanksgiving, Christmas, I guess there’s just like always stuff around, so I still eat well, but when I just have like more of same diet, it definitely is good, and my blood sugars are much better.*
**Theme 2: Impact of diabetes technologies on way of eating and prandial behaviors**
Maladaptive prandial behaviors
6C: *My daughter definitely has a sweet tooth,… and I feel like in a way, she’s felt like she couldn’t get a lot of that stuff in the past, and with [AID system] now, she can be a little less careful and still come up with like about a 6.9% A1c [HbA1c], which is what her A1c [HbA1c] was last time…. [In addition,] part of the relief I get from having the [AID system] is that like, you know, if she forgets [to cover for meals], it’s gotten to be less of a problem.*
1P: *…pre-[AID system], I probably wouldn’t have even eaten [ice cream] because I wouldn’t have corrected, and I would have just [woken] up with a super-high blood sugar…. [AID system is] covering me now because like I know it’s inevitable I’m going to go high, but I won’t not be correcting because I’m asleep. It’ll do it for me.*
Benefits of technology on prandial management
5C: *I think it [technology] has made it easier to manage anything, so being able to see her [CGM] and… those like direct connections between things is really helpful for understanding how different and new foods affect her body…. And then with her pump,… being able to give those microdoses for the lower-carb things and being able to see the actual effect that it has with the [CGM], like those things make it so much easier on a daily basis.*

*Abbreviations:* AID = automated insulin delivery; CGM = continuous glucose monitor; HbA1c = hemoglobin A1c; T1D = type 1 diabetes.

a The letters “C” and “P” in the subject identification number refer to “caregiver” vs“patient” response, respectively.

**Table 4:** Shared Child/Caregiver-Specific Factors Impacting the Relationship Between the Family Unit Encompassing the Child With Type 1 Diabetes and Medical Nutrition Therapy Utilization

Theme 1: Balance between caregiver involvement and child autonomy/emerging independence when it comes to the child’s nutritional management
Challenges
6C^a^: *We know what to do. That just doesn’t mean we do what we’*re *supposed to do. Like she knows exactly what she’s supposed to do, and I’ll like look at her like, “Really, you’*re *going to eat that?” like, but I also get in trouble when I do things like that…. I mean I’m trying to reinforce really good eating habits. I’m just not sure it’s working because, you know, that’s, there’s a part of her that knows what to eat, but that’s not what she wants to eat. It’s not necessarily what she’s going to eat… I mean I can’t control what she eats either way. She’s an adult.*
Collaborations
5C: *I think the communication between her and I and talking about like how things are going to make her feel and things like that and really having open communication is really important so that she can learn, you know, “I feel this way about this, and I feel this way about this,” and kind of separating that is really important, too…. [She’s] really started to make those… healthier choices on her own, and kind of giving her a little bit of that freedom to make choices has definitely helped.*
**Theme 2: Balancing acknowledgment and practice with regard to optimal prandial management**
**(1) Prandial insulin timing**
Challenges:
1P ^a^ : *It’s not always easy to get the 15 min. Like you don’t always know you’*re *going to be eating 15 min ahead of time, so sometimes it’ll be like, “Oh, I’m hungry.” I go get a snack*, *and then I cover as I’m eating, and my blood sugar will end up going high.… [Or] say I’m with my friends and they have some leftover food I’m not expecting it and I want to eat it, so I’ll eat their food…*
Need for variable practices as perceived adaptations:
2C: *…some waffle brands are easy, and we only have to do like a 5-min pre-bolus with a normal carb ratio, and then some waffle brands, we have to like triple the carb ratio and give a 20-min pre-bolus.*
Acknowledgment of importance:
11C: …*I definitely think pre-bolusing before a meal or a snack, as annoying as it can be, and it doesn’t always work, but I think it’s one of the most important things to do with food and nutrition for a type 1 diabetic*.
**(2) Carbohydrate/insulin matching**
Challenges:
7P: …*I was not really bolusing myself for the whole amount of carbs before, which is another reason why my A1c [HbA1c] was really high…. I’ve always been really scared of going low…. [It’s] like a very big fear of mine…*
Need for variable practices as perceived adaptations:
1P: *So I kind of learned, like I’ll have like the same portion for each food, and I’ll kind of know, like even though it’ll say a certain amount on the package, I’ll know how much I should really cover.… [If] it’s something new that I’ve never eaten before, I’ll check the carbs and I’ll kind of estimate off that. Like say my blood sugar [is] on the lower side, then I’ll cover less carbs even though it’s a certain amount. If it’s on the higher side, I’ll cover the right amount and I’ll correct for my blood sugar. [But] if it’s something like I frequently have, like I’ll estimate based on how much I’m going to eat and like what my blood sugar is and like what’s happened with my blood sugar after I ate it in the past.*
Acknowledgment of importance:
11C: *Some of the simpler carbohydrates – …things that are maybe not protein or really heavy in fat – I see like a typical pattern of blood sugars where it will rise and then kind of settle back down, you know, somewhere around that 3-h window, and if it isn’t, then I realize something’s off. You know, maybe we under-bolused. Maybe we over-bolused if she’s low.*
**(3) Meal composition/challenging foods**
Challenges:
10P: …*I feel like even though I am getting the correct amount… of insulin for each meal, I feel like if I am having a bigger meal, my blood sugar tends to go up a little bit more*.… *Let’s say I had a bag of chips, and I gave myself insulin for that, I feel like it would be steady, and then say I had a big chicken sandwich with like fries on the side and a salad and a bigger meal, I think even if I am giving the correct amount of insulin for that meal, I tend to go up, like my blood sugar rises a little bit more.*
Management strategies/substitutions for challenging foods:
8P: …*pizza, for instance, doesn’t always… spike him right away,… so I started to take that 90 g of carbs and just put it over a 3-h period of time*.
9P: *Things like proteins with low-carb other foods, which is mostly fruits and vegetables, I won’t dose for…. I like to have yogurt and strawberries a lot in the morning, and I rarely ever dose for breakfast because it’s like such a low-carb berry,… with like a high-protein yogurt, it just… stabilizes my blood sugar.*
Observed effects of dietary factors on health beyond glycemia:
9C: *If you eat like just like white bread or anything highly processed,… you’*re *not going to feel as good. You’*re *not going to have sustained energy as long*.
**(4) Factors driving decisions regarding approaches to child’s way of eating**
Emphasis on maintaining normalcy/avoidance of dietary restriction and impact on child’s diet quality:
2C: …*so we try really hard not to restrict her because of her diabetes…. We don’t want the diabetes to impact her…. I feel like nutrition is a massive part of all the decisions we have to make, but I feel like unfortunately it also has to sometimes go on the back burner so that I can allow her to still have like a normal childhood.… We don’t want the diabetes to impact her, so like this weekend, we went to a gluten-free expo…, and [patient] literally spent 2 and a half hours eating every single carb-heavy item you can imagine, but she was thrilled because everything was safe for her, and so like knowing that she was going to go high no matter what we did, no matter how we managed it, and just sort of letting that go that there are going to be a couple hours of really poor… blood sugars, it was worth it for her to experience that.*
6P: …*if I really want to eat something that I know might make my blood sugars go a little higher, I still will because I feel like it’s important. I’m not going to like deprive myself of something…*
11C: *You know, she likes a party, and she’d rather eat stuff that’s not as healthy… Definitely her mindset is sugar and sweets and carbs, and I think that’s part of her being a teenager as well, like she just craves that.… [She] doesn’t particularly want to make good choices…*
Concern for the development of disordered dietary habits:
9P: *So like for the first year, I kind of starved myself because people told me that was going to help my blood sugars, so my parents stepped in and made sure I was eating all the time…*
Focus on mindful eating habits and impact on child’s diet quality:
8C: …*for the most part, I try to really give him like a balanced like meal, like protein, fiber, carbs. Like he’ll always have a fruit with his meal, and then the fat’s always in there somewhere… I really try to be cognizant of like the food that goes in his body, and I try to minimize like really junky, processed food because again, you can’t really control where that’s coming from.*
Variable understanding/comfort with managing child’s way of eating:
6P: *I feel like I have like a very good grasp on how to insulin for my food and how to eat portions that I know will like make my blood sugars like not spike and that will just like make me feel good…*
2C: …*what’s been the hardest is that even though we understand her nutritional needs, we understand how insulin acts with different foods in terms of glycemic index, in terms of like digestion with fat and protein, like we know all of those things, so you would think that her CGM line would be nice and straight and flat, but it’s not.*
**Theme 3: Juxtaposition between perception of overall importance of nutrition in T1D care and desire for normalcy**
General perception of emphasizing normalcy/prioritizing diabetes management
2C: *Nutrition is a huge piece that is kind of every single meal. Every single piece of food we put in our mouth, right, we’*re *thinking about how that’s going to impact her blood sugar, but I also just sometimes have to like turn that off…. It’s both, right? It’s so important, and then it has to sometimes not be important.… I think that they should eat cake when there is cake.*
9P: …*you just need to know how much to bolus and you need to know that you have to bolus, otherwise you don’t need to change what you’*re *eating whatsoever*…
Acknowledgment of importance of mindful eating habits/individualization
10C: *I think all kids should eat in moderation and eat pretty healthy,… but I don’t think there’s one way to do it, especially if you have other health things going on*.
Perception of overall important of nutrition in child’s T1D management
6C: *I mean everything revolves around food, and depending on what we eat, it will affect the exercise, will affect the other things.… [So] it’s definitely high up there, and it takes a lot of thought every time you eat anything, so yeah, it’s always top of mind.*
7P: *I think that nutrition is like, like if it’s not the top of the pyramid, I think it’s like the level below it because… no matter what, you’*re *going to have to take insulin, and it doesn’t matter what device you’*re *using, so I think it’s like one of the most important things that you should be like thinking about.*

*Abbreviations:* T1D = type 1 diabetes.

a The letters “C” and “P” in the subject identification number refer to “caregiver” vs“patient” response, respectively.

**Table 5:** Interpersonal Factors Impacting the Relationship Between the Family Unit Encompassing the Child With Type 1 Diabetes and Medical Nutrition Therapy Utilization

General familial factors
**Theme 1: Effect of household composition and inter-caregiver dynamics on approaches to the child’s way of eating**
Nontraditional households/divergent approaches
7C ^a^ : …*our 2 households parent really differently, handle diabetes really differently, handle food really differently, and that is my biggest struggle, and I think that it’s something that a lot of households deal with, whether it is a child who has 2 different houses or 2 very different parents within a household or caregivers like who have wildly different views about what nutrition is, how much they’*re *managing it, pre-bolusing, you know, any of those strategies…. [So] specifically the amount of time pre-bolusing, especially for what we call spikier foods, we’ve had a struggle with consistency between the 2 households that [the patient] lives in, and that’s been challenging.*
Intact households/convergent approaches
9C: …*it’s very rare for one of our family members to eat anything that doesn’t include like protein…. [So] like every meal has protein, so my wife and I participate in all that. Like we do it together, so we make all the food pretty much. You know, we only eat out like once a week for the most part unless we’*re *traveling for hockey, but like they don’t eat fast food. We don’t have chips around. We don’t have a lot of those like high processed-snacks and stuff.*
**Theme 2: Attempts at matching child’s way of eating with that of the rest of the family**
Challenges with matching
8P ^a^ : *Like if my family is eating one thing, like it’s kind of hard to eat something else, right, especially when what they’*re *eating looks so good*…
Successful integration
5C: …*we try to do a healthier snack, and keeping things the same for all my kids definitely helps with the nutrition side of things.… [And] we don’t let any of them gorge on anything, so it’s like, “Okay, your treat is like one piece of candy, a small slice of cake, or a small brownie,” and the portion size is the same for my nine-year-old, my five-year-old, and my almost two-year-old. -*
**Peer-related factors**
**Theme 1: Difficulties surrounding matching child’s way of eating with that of the peers**
7P: …*I look at my friends eating, and like I wish I could just eat freely, you know, but I can’t do that*…
**Theme 2: Challenges with prandial diabetes management in peer settings**
9P: *Not that I was embarrassed. It’s just like I would forget, like we’*re *[patient and friends] just like all eating things and stuff, and I’d be like, “I probably ought to dose for that,” and I would end up dosing afterwards…*

a The letters “C” and “P” in the subject identification number refer to “caregiver” vs“patient” response, respectively.

**Table 6:** Institutional Factors Impacting the Relationship Between the Family Unit Encompassing the Child With Type 1 Diabetes and Medical Nutrition Therapy Utilization

School-related factors
**Theme 1: Variable prandial diabetes management experiences with school staff**
2C ^a^ : …*if we can get the school nurse to pre-bolus, we do, but that doesn’t always happen*.
9P ^a^ : *I’ve had a lot of school nurses tell me I can’t eat food, teachers saying I can’t eat food, and it’s just, I think it all kind of starts with like the wrong perception of type 1 diabetes.… They would make me have lunch… in the nurse’s office, and my teacher would be there. Like they very much so dictated the way my school life was in sixth grade, and that was pretty awful.… I had to meet with them a lot, and it ruined my experience for sixth grade.*
Positive/collaborative staff interactions
8P: …*I tell them [school nurses] 3 things. I tell them what my blood sugar is. I tell them how much I’m planning to give, like how much insulin, and then I tell them how many carbs I’m covering for, so I give them this like little sticky note, and then I go take the injection.… [They] do help a lot, but, you know, it’s mostly me just like doing my own thing.*
**Theme 2: Suboptimal school-related dietary experiences**
Dietary challenges
2C: …*the school food providers don’t have a ton of background in nutrition and an understanding of what kids with type 1 need for a well-rounded meal. School lunch is pretty gross to begin with, but then when you are type 1, you know, offering a juice box with lunch is not a good idea. Offering a popsicle with lunch is not a good idea…*
1P: *Like the school lunches, like they don’t seem like super bad, but clearly it’s unhealthy or something because I always under-cover it, and I always end up shooting up after I eat the school lunches.… [There’s] no like nutritional values anywhere at school. Like they don’t tell you how many carbs are in it. They don’t tell you how much sugar’s in it. They don’t tell you if it’s protein. They just give you it. I just have to figure it out on my own.… [It’s] [also] hard to cover 15 min ahead… because you don’t always know what the school lunch is going to be, so sometimes it’s as you get it, you have to cover.*
Diet-related accommodations
(1) Self- and family-directed accommodations:
7C: …*very early on, they were in a private preschool when they were diagnosed. There was no nurse on site,… and so I tried to figure out the snacks that were very, very low carb or no carb that I could send with them so that we would limit the amount of insulin injections they would need…*
2P: …*usually instead of like at school, I’ll just bring like a sandwich, which is still like a meal, but like just like healthier food that is good.*
(2) School-related accommodations:
3P: *Well, my school has like this lunch menu, and it has it in like the nurse’s office, and it’s like a lunch menu, but it has like all the carbs listed below for each thing, so it’s perfect*, *so I can just like see what I’m having, then like count the carbs for it and then just dose for it.*

a The letters “C” and “P” in the subject identification number refer to “caregiver” vs“patient” response, respectively.

**Table 7:** Community/Societal Factors Impacting the Relationship Between the Family Unit Encompassing the Child With Type 1 Diabetes and Medical Nutrition Therapy Utilization

**Factors related to broader community/social settings**
**Theme 1: Variable experiences with prandial diabetes management in social settings**
Challenges
11C ^a^ : …*if we’*re *at a restaurant and she’s chosen a meal that she’s never eaten before, I like to see it first before she just says, “Oh, it’s probably 100 carbs.” I mean that’s a big deal if you’*re *bolusing for 100 carbs, you know, but that’s the reality when you go out to a restaurant that that can be the case, so pre-bolusing isn’t always possible…*
5P ^a^ : …*if you’*re *eating in a social setting, insulin in front of people can be hard. I’ve had rough experiences where people kind of drew attention to me, and it was annoying, or where people saw me doing an injection and it, you know, rubbed them the wrong way.… In the beginning, I was kind of ashamed of it almost, so it made me, you know, kind of hide doing insulin.*
Diet-related accommodations
10P: …*if you’*re *at a fast-food place, you can look usually on their menu, like on their apps, on the food, you can look at the nutrition stuff and has its stuff there*…
Community support/education
10C: *…in my group chat with my friends, if they’ve found something [food] that, you know, they’re not getting a spike from…, “Oh, I found these waffles, no spike, oh, I found these bars, no spike, oh, carb-free drink,” you know. I was like, “Oh, I’ll have to get it next time I’m at the store.” You know, we share what works for us and what could work for other people.*
**Societal factors**
**Theme 1: Nutritional challenges stemming from easier access to foods of lower dietary quality, often in the setting of cultural influences**
Dietary challenges
6P: …*I feel like the American diet and what like an average American consumes is not like good for anyone.… I feel like our society, like the normal diet is definitely like not the same as some other cultures which are healthier and which would not have as much of an effect on blood sugars versus I feel like the average American diet is just very carby and like carbs that aren’t necessarily like nutrient-dense or that great for you or your blood sugars.*
**Theme 2: Potential impact of sociodemographic inequities on MNT utilization**
6C: *I think it’s an individual decision [whether a child with type 1 diabetes should change their way of eating following the diagnosis]. I think every parent, depending on their relationship with food, how much money they have, what they can afford to do – like there’s a lot of like everything being in packages is, you know, a more expensive way to eat.… Expensive foods [however] tend to work better…*

*Abbreviations:* MNT = medical nutrition therapy.

a The letters “C” and “P” in the subject identification number refer to “caregiver” vs“patient” response, respectively.

## Highlights

- Medical nutrition therapy (MNT) in pediatric type 1 diabetes (T1D) is underutilized, however, the reasons for this are not well understood
- Underutilization of MNT can lead to suboptimal glycemic and metabolic outcomes in children with T1D
- Utilizing the socioecological framework and semi-structured interviews with both patients and caregivers, this study uncovered important themes related to barriers to MNT utilization in pediatric T1D that spanned 5 domains (individual, shared child/caregiver-specific, interpersonal, institutional, and community/societal)
- The central theme was the juxtaposition between perceived importance of MNT in pediatric T1D management and its actual implementation
- Factors mediating the complex relationship between perception of MNT importance and its utilization included challenges with adjustment to dietary changes post-T1D diagnosis, the impact of diabetes technologies on prandial practices, balance between caregiver involvement and emerging patient independence, emphasis on normalcy, attempts to match child’s way of eating with that of others, school-based staff-related/dietary experiences, and cultural/sociodemographic considerations

## Clinical Relevance

This study enhanced our understanding of the barriers underlying optimal medical nutrition therapy (MNT) utilization in pediatric type 1 diabetes that will inform future initiatives aimed at improving MNT uptake. As MNT is integral to pediatric type 1 diabetes management, with documented glycemic and metabolic benefits, this area of research is important to help optimize patient outcomes.

## Abbreviations:

ADA: American Diabetes Association
AID: automated insulin delivery
BCH: Boston Children’s Hospital
CGM: continuous glucose monitor
HbA1c: hemoglobin A1c
MNT: medical nutrition therapy
RD: registered dietitian
T1D: type 1 diabetes
