# Supplementary material for: Qualitative Analysis of Caregiver and Patient Experiences With and Barriers to Medical Nutrition Therapy Utilization in Pediatric Type 1 Diabetes
Source: Endocr Pract. Author manuscript; Available in PMC 2026 Jan 6. (PMC12768430; doi:10.1016/j.eprac.2025.11.005)
Supplement: MMC2 [file NIHMS2125166-supplement-MMC2.docx]

**T1D Nutrition Study Patient Semi-Structured Interview Guide**

1. **Review/sign consent form:**

As the participant and caregiver(s) arrive on Zoom, display the most recent IRB-approved copy of the informed consent/assent form, which the participant and caregiver(s) would’ve received ahead of time via secure email. Review the consent form with the participant and caregiver(s), answering any questions they may have and affording them sufficient time to consider whether to participate. Assess the participant and caregiver(s)’ comprehension by asking open-ended questions such as: “What more would you like to know?”; “To make sure you understand what is expected of you, would you tell me in your own words what you think we are asking you to do?”; “What are the potential benefits of participation to you?”; and/or “What are the potential risks of participation to you?”. After all questions have been answered, obtain the required electronic signatures, and email the copy of the signed consent form to the participant and caregiver(s) to keep. After consent/assent form is assigned, the caregiver(s) will be asked to leave the Zoom meeting.

1. **Introduction (5 minutes)**

**Welcome:**

Thank you so much for coming today and for your willingness to participate in this study.

**Background:**

Our research team at *** wants to learn about how children with type 1 diabetes think about nutrition and what role it plays in their blood sugar control and health. We want to hear about your honest thoughts, experiences, and difficulties when it comes to managing your way of eating as part of your diabetes care. We hope that findings from this study will help us come up with better ways to help take care of children with type 1 diabetes, especially when it comes to nutrition.

**Disclosures:**

- We will be recording today’s session so that we can make sure we capture all your comments. We are not doing this for the purpose of identifying who said what. Instead, we’re interested in making sure that we know what you said.
- You may also see a member of the research team taking notes. Again, this is only because we want to ensure that we remember all your comments. Any notes we take will not be used to identify you in any way.
- When we write our report, no one’s name will ever be used. The report that we write will be used to help develop ways to improve nutritional care for children with type 1 diabetes.

**Ground Rules:**

- We want your honest opinions and reactions. There are no right or wrong answers.
- If there is any question you don’t want to answer, that’s okay. You don’t have to talk about anything you’re not comfortable discussing. We can skip that question.
- Questions?

**Prompt for Interview Start:**

We are now ready to begin the interview. I will start the recording.

1. **Interview:**

**General questions about the child’s type 1 diabetes history (5 minutes):**

- Before we get to questions related to diabetes and nutrition, I would love to hear more about you, including your lifestyle and what activities you like to do. Anything that you want to share with us.
  - *Prompts:*
    - What grade are you in at school, and what is school like for you?
    - Tell me a little bit about the family structure, e.g., are there other children in the home?
- Tell us anything you would like to share about your diabetes story.
  - *Prompts:*
    - What happened that led to the diagnosis?
    - How were you diagnosed?
- How has your overall diabetes control been within the past year?
- Have you or your diabetes team had any other concerns about your health within the past year (e.g., weight gain or weight loss, issues with cholesterol levels)?

**General thoughts about the role of nutrition in a child’s type 1 diabetes care (15 minutes):**

- What comes to mind when you think about the effects that a child’s food choices and eating habits have on their diabetes control?
- What effects do different types and amounts of food have on blood sugars in a child with diabetes?
  - *Prompts:*
    - Do all carbohydrates have the same effect?
    - What effects do foods that are high in fat (e.g., cheese, pizza) have?
    - What effects do foods that are high in protein (e.g., beef, chicken, fish) have?
    - What effects do meal sizes have?
- Tell us what you think about the role and importance of nutrition in a child’s diabetes care.
  - *Prompt:* Do you think children with diabetes should eat differently than children without diabetes?
- How do the role and importance of nutrition compare to other aspects of a child’s diabetes care?
  - *Prompts:*
    - Insulin
    - Devices (e.g., continuous glucose monitors, insulin pumps, hybrid closed-loop systems)
    - Exercise/physical activity
    - Support for mental health and diabetes-related stress
    - Meeting other kids with diabetes (peer support groups, diabetes camps)
- Do you think a child with type 1 diabetes should change their way of eating following the diagnosis?
  - IF YES:
    - Why?
    - In what ways?
  - IF NO:
    - Why not?
- What should children with type 1 diabetes be eating?
  - *Prompt:* Do you think there’s one perfect way of eating that all children with type 1 diabetes should follow?
- What is the role of the dietitian/nutritionist in the care of a child with type 1 diabetes?
- How often do you think a child with type 1 diabetes should meet with a dietitian/nutritionist?

**Patient-specific nutrition-related questions (15 minutes):**

- How do you manage your diabetes (i.e., through injections, regular pump, or special pump that pairs with the continuous glucose monitor, such as the Omnipod 5, Tandem t;slim X2 with Control-IQ, or Medtronic 670G or 770G)?
  - IF “regular pump” or “special pump”:
    - Tell us how technology may have affected your relationship with food/nutrition.
- How do you and/or your caregivers figure out how much insulin to give for your meals (e.g., count carbohydrates, estimate carbohydrates based on prior experiences, take into account the fat and protein content of foods, have set insulin doses for certain foods or meal sizes)?
  - *Prompts:*
    - Do you usually do this by yourself, or do you get help?
    - Has this approach changed over time?
      - *[IF YES]* Please tell us more.
- When do you typically receive your mealtime insulin in relation to eating (e.g., before meals, during meals, after meals, different depending on the situation)?
- Do you have any medical conditions that affect what you can eat (e.g., celiac disease/gluten insensitivity, certain food allergies)?
- Did you follow any specific diet(s) before their diabetes diagnosis (e.g., low carbohydrate, vegetarian)?
- Do you currently follow any specific diet(s) (e.g., low carbohydrate, vegetarian)?
- Have you changed or tried to change your way of eating in any way since your diabetes diagnosis?
  - IF YES:
    - In what ways?
    - What strategies have you used to make these changes work for you?
    - What has been your experience with these changes?
      - *Prompt:* Have you face any difficulties while changing or trying to change your way of eating?
  - If NO:
    - Do you want to change your way of eating?
      - *[IF YES]* In what ways?
- We know how difficult it is to manage the way a child with type 1 diabetes eats. We want to try to understand what you’re doing in real life, even things that might not come out during your medical visits. There’s absolutely no judgment. With that in mind, tell us about your current way of eating.
  - *Prompt:* Do you consider your way of eating healthy?
- Many children with type 1 diabetes have certain foods that are hardest for their blood sugars. What are some of the foods that have been the hardest for your blood sugars?
- Do you ever change what you do about your food depending on whether your blood sugars are low or high (e.g., eat uncovered snack/meal if low, delay eating if high)?
- Some children with type 1 diabetes eat uncovered meals or snacks (meaning that they don’t receive insulin with their food). Tell us about any scenarios when you might eat uncovered meals or snacks.
- Tell us about what you do about your nutrition when it comes to exercise/physical activity.
- Have you noticed any effects of your food choices and eating habits on your blood sugars?
  - *[IF YES]* What effects have you noticed?
- Do you think you have a good understanding of how nutrition affects your diabetes control?
- Do you think you have a good understanding of how to manage your way of eating?
  - IF YES:
    - Do you think having a good understanding alone is enough in making healthy choices?
    - Do you feel comfortable managing your way of eating?
- Do you think about your way of eating when making decisions about your diabetes care?
  - *Prompts:*
    - If your blood sugars are often high, do you think about changing what you’re eating?
    - How does changing your way of eating compare to changing other aspects of your diabetes care (e.g., insulin doses, activity levels)?
- Some children with type 1 diabetes have difficulties when trying to manage their way of eating (e.g., not liking certain foods, not wanting to eat differently from their family members and/or friends, arguing with caregivers about food choices). Have you encountered any difficulties in managing your way of eating?
  - IF YES:
    - Please tell us more about these difficulties.
      - *Prompts:*
        - When it comes to family
        - When it comes to friends/peers
        - When it comes to school
    - How have you tried to overcome these difficulties?

**Experiences with nutritional topics and advice encountered during visits with dietitian/nutritionist** **and medical providers (10 minutes):**

- Have you attended any visits with a dietitian/nutritionist since your diabetes diagnosis?
  - IF YES:
    - Please tell us about these experiences.
      - *Prompts:*
        - What topics were covered?
        - What advice did they give?
        - How helpful has their advice been?
        - Have you encountered any difficulties applying their advice to your day-to-day life?
        - Have you noticed any effect of this advice on your diabetes control and health?
  - IF NO:
    - Why not?
- Does your diabetes nurse educator or doctor talk to you about going to see the dietitian/nutritionist?
  - IF YES:
    - Which provider(s) spoke to you about this (i.e., diabetes nurse educator, diabetes doctor, or both)?
    - What have those experiences been like?
- Have your diabetes nurse educator or diabetes doctor discussed any nutritional topics or provided any dietary advice during your medical visits?
  - IF YES:
    - Which providers spoke to you about this (i.e., diabetes nurse educator, diabetes doctor, or both)?
    - What topics were covered?
    - What advice did they give?
    - How helpful has their advice been?
    - Have you encountered any difficulties applying their advice to your day-to-day life?
    - Have you noticed any effect of this advice on your diabetes control and health?
  - IF NO:
    - We are really interested in learning how to help our diabetes nurse educators and diabetes doctors talk about nutrition topics with our patients and families. Is this something you’re interested in hearing more about from your providers?
      - IF YES:
        - What specific topics do you want to talk more about?
        - What suggestions do you have for us on how we can make this happen?

**Additional information:**

- Before we finish, is there anything else about the role of nutrition in diabetes care that we haven’t asked you about that you’d like to share or comment on?

I will now stop the recording.

1. **Closing (5 minutes)**

Thank you so much for your time and help. Your responses gave us very useful information that will help us understand how children with type 1 diabetes think about the role of nutrition in their diabetes care. To gather more information on this topic, we will now appreciate you taking a survey to help us gather even more information on the topics covered during today’s interview. As you complete the survey, we would like your honest feedback along the way, including if there’s anything that’s not clear or that you do not understand. This will allow us to make the survey even better before we send it out to all children ages 12 to 17 years who have had type 1 diabetes for at least a year followed by the *** Diabetes Program. We will also use what we learn to develop a survey for diabetes nurse educators and diabetes doctors to learn how they approach nutrition in children with type 1 diabetes. Combined findings from the interviews and the surveys will hopefully allow us to come up with better nutritional strategies to help manage type 1 diabetes in children.
