# Supplementary material for: Qualitative Analysis of Caregiver and Patient Experiences With and Barriers to Medical Nutrition Therapy Utilization in Pediatric Type 1 Diabetes
Source: Endocr Pract. Author manuscript; Available in PMC 2026 Jan 6. (PMC12768430; doi:10.1016/j.eprac.2025.11.005)
Supplement: MMC1 [file NIHMS2125166-supplement-MMC1.docx]

**T1D Nutrition Study Caregiver Semi-Structured Interview Guide**

1. **Review/sign consent form:**

As the participant arrives on Zoom, display the most recent IRB-approved copy of the informed consent form, which the participant would’ve received ahead of time via secure email. Review the consent form with the participant, answering any questions they may have and affording them sufficient time to consider whether to participate. Assess the participant’s comprehension by asking open-ended questions such as: “What more would you like to know?”; “To make sure you understand what is expected of you, would you tell me in your own words what you think we are asking you to do?”; “What are the potential benefits of participation to you?”; and/or “What are the potential risks of participation to you?”. After all questions have been answered, obtain the required electronic signature, and email the copy of the signed consent form to the participant to keep.

1. **Introduction (5 minutes)**

**Welcome:**

Thank you so much for coming today and for your willingness to participate in this study.

**Background:**

Our research team at *** wants to learn about how caregivers of children with type 1 diabetes approach the role of nutrition in a child’s diabetes care and what effects dietary factors have on blood sugar control. We also want to hear about your thoughts, experiences, and challenges when it comes to nutritional management of your own child’s type 1 diabetes. We hope that findings from this study will be used to design effective strategies and interventions aimed at improving medical practice.

**Disclosures:**

- We will be recording today’s session so that we can make sure we capture all your comments. We are not doing this for the purpose of identifying who said what. Instead, we’re interested in making sure that we know what you said.
- You may also see a member of the research team taking notes. Again, this is only because we want to ensure that we remember all your comments. Any notes we take will not be used to identify you in any way.
- When we write our report, no one’s name will ever be used. The report that we write will be used to help develop ways to improve nutritional care for children with type 1 diabetes.

**Ground Rules:**

- We want your honest opinions and reactions. There are no right or wrong answers.
- If there is any question you don’t want to answer, that’s okay. You don’t have to talk about anything you’re not comfortable discussing. We can skip that question.
- Questions?

**Prompt for Interview Start:**

We are now ready to begin the interview. I will start the recording.

1. **Interview:**

**General questions about the child’s type 1 diabetes history (5 minutes):**

- Before we get to questions related to diabetes and nutrition, I would love to hear more about your child, including their lifestyle and what activities they like to do. Anything that you want to share with us.
  - *Prompts:*
    - What grade are they in at school, and what is school like for them?
    - Tell me a little bit about the family structure, e.g., are there other children in the home?
- Tell us anything you would like to share about your child’s diabetes story.
  - *Prompts:*
    - What happened that led to the diagnosis?
    - How was your child diagnosed?
- How has your child’s overall diabetes control been within the past year?
- Have you or your child’s diabetes team had any other concerns about your child’s health within the past year (e.g., weight gain or weight loss, issues with cholesterol levels)?

**General thoughts about the role of nutrition in a child’s type 1 diabetes care (15 minutes):**

- What comes to mind when you think about the effects that a child’s food choices and eating habits have on their diabetes control?
- What effects do different types and amounts of food have on blood sugars in a child with diabetes?
  - *Prompts:*
    - Do all carbohydrates have the same effect?
    - What effects do foods that are high in fat (e.g., cheese, pizza) have?
    - What effects do foods that are high in protein (e.g., beef, chicken, fish) have?
    - What effects do meal sizes have?
- Tell us what you think about the role and importance of nutrition in a child’s diabetes care.
  - *Prompt:* Do you think children with diabetes should eat differently than children without diabetes?
- How do the role and importance of nutrition compare to other aspects of a child’s diabetes care?
  - *Prompts:*
    - Insulin
    - Devices (e.g., continuous glucose monitors, insulin pumps, hybrid closed-loop systems)
    - Exercise/physical activity
    - Support for mental health and diabetes-related stress
    - Meeting other kids with diabetes (peer support groups, diabetes camps)
- Do you think a child with type 1 diabetes should change their way of eating following the diagnosis?
  - IF YES:
    - Why?
    - In what ways?
  - IF NO:
    - Why not?
- What should children with type 1 diabetes be eating?
  - *Prompt:* Do you think there’s one ideal way of eating for all children with type 1 diabetes?
- What is the role of the dietitian/nutritionist in the care of a child with type 1 diabetes?
- How often do you think a child with type 1 diabetes should meet with a dietitian/nutritionist?

**Patient-specific nutrition-related questions (15 minutes):**

- How does your child manage their diabetes (i.e., through injections, regular pump, or special pump that pairs with the continuous glucose monitor, such as the Omnipod 5, Tandem t;slim X2 with Control-IQ, or Medtronic 670G or 770G)?
  - IF “regular pump” or “special pump”:
    - Tell us how technology may have affected your child’s relationship with food/nutrition.
- How do you and/or your child figure out how much insulin to give for your child’s meals (e.g., count carbohydrates, estimate carbohydrates based on prior experiences, take into account the fat and protein content of foods, have set insulin doses for certain foods or meal sizes)?
  - *Prompts:*
    - Does your child usually do this by themselves, or do they get help?
    - Has this approach changed over time?
      - *[IF YES]* Please tell us more.
- When does your child typically receive their mealtime insulin in relation to eating (e.g., before meals, during meals, after meals, different depending on the situation)?
- Does your child have any medical conditions that affect what they can eat (e.g., celiac disease/gluten insensitivity, certain food allergies)?
- Did your child follow any specific diet(s) before their diabetes diagnosis (e.g., low carbohydrate, vegetarian)?
- Does your child currently follow any specific diet(s) (e.g., low carbohydrate, vegetarian)?
- Have you changed or tried to change the way your child eats since their diabetes diagnosis?
  - IF YES:
    - In what ways?
    - What strategies have you used to make these changes work for you and your child?
    - What has been your child’s experience with these changes?
      - *Prompt:* Have you encountered any challenges while changing or trying to change your child’s way of eating?
  - If NO:
    - Do you want to change your child’s way of eating?
      - *[IF YES]* In what ways?
- We know how difficult it is to manage the way a child with type 1 diabetes eats. We want to try to understand what you’re doing in real life, even things that might not come out during your child’s medical visits. There’s absolutely no judgment. With that in mind, tell us about your child’s current way of eating.
  - *Prompt:* Do you consider your child’s way of eating healthy?
- Many children with type 1 diabetes have certain foods that are hardest for their blood sugars. What are some of the foods that have been the most challenging for your child’s blood sugars?
- Do you ever change what you do about your child’s food depending on whether their blood sugars are low or high (e.g., give uncovered snack/meal if low, delay mealtime if high)?
- Some children with type 1 diabetes eat uncovered meals or snacks (meaning that they don’t receive insulin with their food). Tell us about any scenarios when your child might eat uncovered meals or snacks.
- Tell us about how you approach your child’s nutrition when it comes to exercise/physical activity.
- Have you noticed any effects of your child’s food choices and eating habits on their blood sugars?
  - *[IF YES]* What effects have you noticed?
- Do you think you have a good understanding of how nutrition affects your child’s diabetes control?
- Do you think you have a good understanding of how to manage your child’s way of eating?
  - IF YES:
    - Do you think having a good understanding alone is enough in helping your child make healthy choices?
    - Do you feel comfortable managing your child’s way of eating?
- Do you think about your child’s way of eating when making decisions about their diabetes care?
  - *Prompts:*
    - If your child’s blood sugars are constantly high, do you think about changing what they eat?
    - How does changing your child’s way of eating compare to changing other aspects of their diabetes care (e.g., insulin doses, activity levels)?
- Some families of children with type 1 diabetes encounter challenges when trying to manage their child’s way of eating (e.g., children may sneak extra snacks, make unhealthy food choices, argue with caregivers about their eating habits, etc.). Have you encountered any challenges in managing your child’s way of eating?
  - IF YES:
    - Please tell us more about these challenges.
      - *Prompts:*
        - When it comes to your child’s family
        - When it comes to your child’s friends/peers
        - When it comes to your child’s school
    - How have you and your child tried to overcome these challenges?

**Experiences with nutritional topics and recommendations encountered during visits with dietitian/nutritionist** **and medical providers (10 minutes):**

- Have you attended any visits with a dietitian/nutritionist since your child’s diabetes diagnosis (do not count the initial education you received at the hospital)?
  - IF YES:
    - Please tell us about these experiences.
      - *Prompts:*
        - What topics were covered?
        - What recommendations did they provide?
        - How helpful have their recommendations been?
        - Have you encountered any challenges implementing their recommendations?
        - Have you noticed any impact of these recommendations on your child’s diabetes control and health?
  - IF NO:
    - Why not?
- Does your child’s diabetes nurse educator or doctor talk to you about going to see the dietitian/nutritionist?
  - IF YES:
    - Which provider(s) spoke to you about this (i.e., diabetes nurse educator, diabetes doctor, or both)?
    - What have those experiences been like?
- Have your diabetes nurse educator or diabetes doctor discussed any nutritional topics or provided any dietary recommendations during your child’s medical visits?
  - IF YES:
    - Which providers spoke to you about this (i.e., diabetes nurse educator, diabetes doctor, or both)?
    - What topics were covered?
    - What recommendations have they provided?
    - How helpful have these recommendations been?
    - Have you encountered any challenges implementing the recommendations?
    - Have you noticed any impact of these recommendations on your child’s diabetes control and health?
  - IF NO:
    - We are really interested in learning how to help our diabetes nurse educators and diabetes doctors talk about nutrition topics with our patients and families. Is this something you’re interested in hearing more about from your providers?
      - IF YES:
        - What specific topics do you want to talk more about?
        - What suggestions do you have for us on how we can make this happen?

**Additional information:**

- Before we finish, is there anything else about the role of nutrition in diabetes care that we haven’t asked you about that you’d like to share or comment on?

I will now stop the recording.

1. **Closing (5 minutes)**

Thank you so much for your time and input. Your responses provided invaluable information to help us understand how caregivers of children with type 1 diabetes think about the role of nutrition in a child’s diabetes care. To gather more information on this topic, we will now appreciate your participation in a survey that we had designed to complement the topics covered during the interview. As you complete the survey, we would like your honest feedback along the way, including if there’s anything that’s not clear or that you do not understand. This will allow us to refine the survey before sending it out to all caregivers of children with type 1 diabetes for at least a year followed by the *** Diabetes Program. We will also use these insights to develop a survey for diabetes medical providers (nurse educators and doctors) to learn how they approach nutrition in children with type 1 diabetes. Combined findings from the interviews and the large-scale surveys will hopefully allow us to develop optimal nutrition strategies for the management of type 1 diabetes in children.
